# Research beyond Walls: State University of New York (SUNY) Eye Institute

**Published:** 2012-01

**Authors:** William J Brunken, Barry E Knox

**Affiliations:** 1 SUNY Eye Institute, Departments of Ophthalmology and Cell Biology, SUNY Downstate Medical Center, Brooklyn, NY, USA; 2 SUNY Eye Institute, Departments of Neuroscience and Physiology and Ophthalmology, SUNY Upstate University, Syracuse, NY, USA

## OVERVIEW

Ophthalmic research has developed into a multidisciplinary enterprise that requires the collaboration of basic and clinical scientists to understand and treat diseases that affect virtually every step in vision: from the entrance of light into the eye, through transduction in the retina, to higher processing in the visual cortex. It is a challenge to create and support a critical mass of diverse researchers together in one location. A novel solution to this dilemma has taken form at the State University of New York (SUNY, www.suny.edu), where 5 geographically distributed vision research groups have united to form the multidisciplinary research institute, i.e. SUNY Eye Institute (SEI, www.sunyeye.org).

SEI, one of the largest vision research groups in the US, represents a novel and exciting model by integrating the complementary resources of the four medical schools in the State University of New York system and the College of Optometry into a single research institute. This cooperative venture combines the three core constituencies of vision research - basic, ophthalmic, and optometric sciences - into a single research enterprise. This model represents a paradigm shift, yet built on traditional academic infrastructure, using communication technology to generate a state-wide wall-less institute. Thus, SEI serves a broad and diverse constituency in New York, the third most populous state in the United States.

The SEI mission is to integrate complementary strengths of the SUNY campuses into a single institute to enhance research and training in clinical and basic visual sciences. The goals are as follows:

– To promote and foster collaborative research interactions among SEI scientists.
– To establish state-of-the-art eye research core facilities to serve and augment SEI researchers.
– To recruit basic and clinical investigators into eye and vision research.
– To develop pre- and post-doctoral training programs in eye and vision research across the five campuses.

## HISTORY

**Figure 1.**
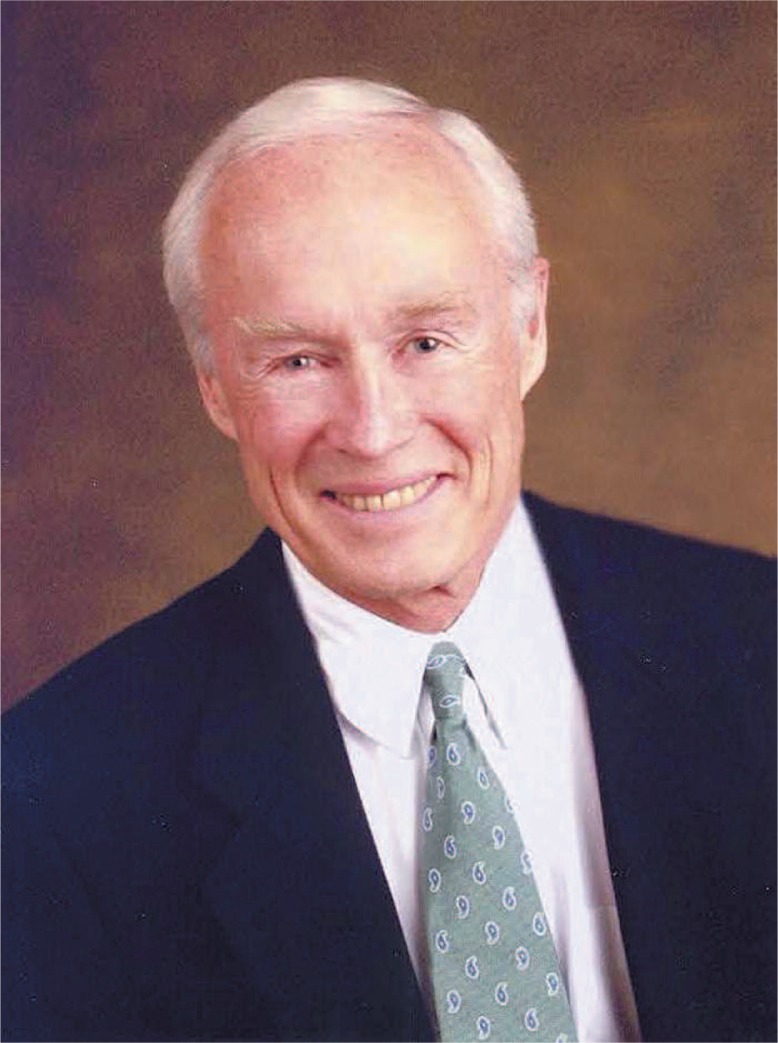
Dr. Robert B. Barlow, Jr. (1939 –2009). Professor and co-founder, Center for Vision Research, Department of Ophthalmology, SUNY Upstate Medical University, Syracuse, NY (Passaglia (2010) J. Exp. Biol. 213, 1397). Dr. Barlow was instrumental in founding the SEI in 2007.

SEI was created by faculty initiative led by Dr. Robert Barlow (SUNY Upstate, Fig. 1). In November 2007, a collaborative agreement between ophthalmology groups at SUNY Downstate Medical Center and SUNY Upstate Medical University, the two stand-alone SUNY medical schools, was begun to form a limited translational research collective. In May 2008, the collective had grown to include SUNY Buffalo, the College of Optometry and SUNY Stony Brook. The SUNY Eye Institute was officially established in January 2009. Since then, the institute has developed a formalized structure and mission. A highlight of SEI activities is the annual workshop with keynote presentations, a selection of which is presented following this article.

## STRUCTURE

Currently, forty-five research-active SEI faculty members direct, co-direct, or collaborate in laboratory-based vision research programs, dealing with over thirty-three independently supported projects. In addition, a number of faculty members direct several shared training programs and clinical trials. For example, the first center for pediatric ocular pharmacology in the US was established. Headed by Dr. Jacob Aranda, it combines the resources of neonatalogists and ophthalmologists in New York City and is focused on retinopathy of prematurity.

The potential clinical and translational impact of the SEI is exciting. The three New York City metropolitan centers have over 275,000 patient visits annually with a rich demographic diversity reflecting the catchment area of metropolitan New York while the two upstate centers have an additional 40,000 patient visits. SUNY Stony Brook has a long track record of epidemiologic studies and clinical trials serving as the coordinating center for the SEI. Investigators currently collaborate across the five campuses using multiple internet modalities and traditional mechanisms.

## SCIENTIFIC THEMES

The scientific expertise of SEI investigators encompasses all major biomedical disciplines, including biochemistry, genetics, bioengineering, cell, molecular and developmental biology, epidemiology, immunology, neurobiology, metabolism, pharmacology and physiology. Research concentrations of our investigators can be grouped as follows.

– Repair, remodeling, and regeneration.
– Developmental biology of the eye and inherited eye defects.
– Epidemiology, clinical trials, and clinical outcomes.
– Metabolism and neovascularization.
– Pathophysiology of ocular disease.
– CNS processing and low vision.

## IMPLEMENTATION

The SEI administrative structure is designed to build and nurture the collaborative concept of the institute, guided by a steering committee with representatives from each institution. SEI activities have focused on promoting collaboration, including: (i) shared web-cast SEI seminars, (ii) annual SEI ARVO meetings (iii) annual weekend workshops; (iv) sponsored pilot projects; and (v) group lab/data meetings via both high-definition teleconference sites or via computer-to-computer connectivity.

## EDUCATION

SEI is committed to crossing traditional disciplinary boundaries with joint appointments in both basic science and clinical departments. Training is a core-strength at all SEI institutions. SEI faculty actively participate in undergraduate and graduate clinical training, as well as undergraduate, graduate and post-doctoral training in basic (translational) sciences. Currently, there are 46 ophthalmology and 31 optometry residents, 28 post-doctoral fellows, 11 medical, and 33 graduate (Ph.D., M.D./Ph.D., M.S.) students training in our combined programs. One of the most important aspects of SEI is mentoring younger investigators, from students to junior faculty. The pool of senior mentors is deep across all five institutions, affording unique advantages to traditional brick-and-mortar institutions. By design, the core facilities afford members of the community access to a wide range of expertise, technology and advice.

## CONCLUSION

**Figure 2.**
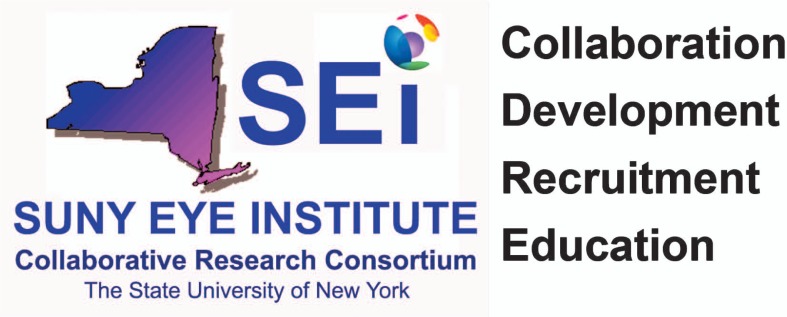
Mission of the SUNY Eye Institute

The establishment of SEI and its core laboratories represent a unique and exciting direction in vision research (Fig. 2). Each participating institution brings unique strengths and specialties to the SEI. This is reflected, for example, in the specialized centers that exist for the application of bioinformatics/proteomics at Buffalo, the generation and analysis of genetically manipulated animals in Syracuse, and epidemiological studies in Stony Brook. These are coupled with strong translational interest in neurodegenerative and vascular disease at Downstate and higher cortical processing and scientific computing at SUNY Optometry. This diversity in research programming and expertise will not only facilitate innovation and collaboration but will hopefully advance more rapidly the understanding of eye function, diseases and their treatment. Moreover, this wall-less institute represents one way to the future as former geographic boundaries are becoming less important to the next generation of scientists in an interconnected world.

